# Views and Experiences of Dementia in People With Intellectual Disabilities: A Systematic Review of Qualitative Research

**DOI:** 10.1111/jir.13227

**Published:** 2025-03-16

**Authors:** Joanna Carter, Aimee Spector, Afia Ali, Amelia McFeeters, Sarah Butt, Georgina Charlesworth

**Affiliations:** ^1^ Research Department of Clinical, Educational & Health Psychology University College London London UK; ^2^ Wolfson Institute of Population Health Queen Mary University of London London UK; ^3^ Independent Researcher London UK

**Keywords:** dementia, intellectual disability, qualitative research, systematic review

## Abstract

**Background:**

It is important to hear the perspectives of people with intellectual disabilities on dementia. This review aimed to explore views and experiences of dementia from the perspective of people with intellectual disabilities and methodologies enabling people with intellectual disabilities and dementia to participate in qualitative research.

**Methods:**

Studies were identified in database searches, along with reference and citation searches. Qualitative data were reviewed using thematic synthesis and risk of bias assessed using the Critical Appraisal Skills Programme (2018). Methodologies used to include participants with intellectual disabilities and dementia were reviewed.

**Results:**

Findings from 11 studies, with a total of 47 participants, highlighted loss of ability, relationships and connection associated with dementia, counteracted by support from others, and maintenance of a sense of self through choice, relational connection and competence. A range of methodologies were identified to enable participants with intellectual disabilities and dementia to participate in research.

**Conclusions:**

This review highlights emerging, albeit demographically limited, qualitative research in this field. It suggests ways to build on this including methodologies to facilitate inclusion of people with intellectual disabilities and dementia in further research.

## 1 Background

It is acknowledged that hearing first‐hand experiences, not solely proxy reports, helps to better understand dementia (Wilkinson 2002), with increasing research exploring the personal accounts of people with dementia in order to shape approaches to care and conceptual frameworks (Górska et al., 2017; Patterson et al. 2018). People with intellectual disabilities are also increasingly involved in research exploring their perspectives (Beail and Williams 2014); however, consideration of perspectives are lagging in relation to the topic of dementia (Watchman et al. 2019). This is important given the increasing complexity of age‐related needs for people with intellectual disabilities as they live longer (Sheerin et al. 2024), with dementia impacting physical ability, communication, independence, health needs and access to networks (Jacobs et al. 2023).

There are barriers to people with intellectual disabilities with or without dementia participating in research, including difficulties around consent, capacity, communication, level of cognition, as well as researcher views, skills and experience (Crook et al., 2016; Shepherd et al. 2022; Stalker 1998). However, accessible methodologies and appropriate adjustments can overcome challenges and enable people with cognitive disabilities to participate in research (Sheth 2019a; Wilkinson 2002). There is a need to identify data collection methods to facilitate the gathering of perspectives of people with intellectual disabilities and dementia (Watchman et al. 2019).

It is important to further understand these experiences through first‐hand accounts, to identify what research has been undertaken and identify methods used to include people with intellectual disabilities and dementia in research. A recent review explored perspectives of people with intellectual disabilities and dementia and their carers (Jacobs et al. 2023); however, two of the studies solely relied on proxy reports whose perspectives may differ from the person with intellectual disabilities (Watchman et al. 2019).

Although recommended by attendees of the international summit on intellectual disability and dementia (Watchman et al. 2019), no systematic review to date has explored the views and experiences of dementia from the perspective of people with intellectual disabilities both with and without dementia. This review therefore aimed to summarise qualitative research exploring views and experiences of people with intellectual disabilities in regard to dementia; and methodologies that enable participation of people with intellectual disabilities and dementia in qualitative research. This research also focused solely on qualitative research to be able to gather the personal stories identified as important in previous research (Górska et al., 2017).

## 2 Methodology

Searches in PsycINFO, Medline and Web of Science were undertaken using four concepts: intellectual disability, dementia, qualitative research and views/experiences, with keywords, outlined in Data S1. Reference lists and citing articles of included studies were reviewed.

The SPIDER tool (Cooke et al. 2012) structured eligibility criteria (Table 1), which included some grey literature and articles in other languages were considered if a suitable translation could be acquired. After searches, records were downloaded into EndNote (version X9). The first author carried out automatic and manual de‐duplication, screened titles, and abstracts for eligibility, then reviewed the full text of the remaining reports. A second researcher applied eligibility criteria to 10% of titles and abstracts and 50% of included papers. Differences in inclusion of studies were discussed.

**TABLE 1:** SPIDER table of study inclusion and exclusion criteria.

Area	Inclusion criteria	Exclusion criteria
Sample	People with ID with or without dementia	People without ID
Phenomenon of interest	Studies exploring experiences and views of dementia from the perspective of people with ID Studies exploring views and experiences of people with ID and dementia on any topic	Studies only examining the perspectives of staff or caregivers
Design	Qualitative or mixed‐methods studies reporting primary qualitative data (e.g., through participant observation and focus groups or interviews)	Studies only reporting quantitative data
Evaluation	Qualitative analysis of views and experiences of people with ID	Only quantitative methods
Research type	Peer‐reviewed journal articles Dissertations and theses	Systematic reviews Protocols Editorials Opinion pieces

The first author used the Critical Appraisal Skills Programme (CASP; 2018) Qualitative Studies Checklist to evaluate included studies. The second researcher rated half the studies using CASP and compared scores to measure agreement in applying the checklist.

The first author completed data extraction using a spreadsheet designed to collate participant demographics, methodologies and results.

Qualitative findings of studies were analysed using thematic synthesis (Thomas and Harden 2008). Text was imported into NVivo and line by line coding of all text in the findings was conducted to search for concepts, in line with previous studies (McMahon et al. 2022; Thomas and Harden 2008). In order to ascertain any differences between participants with and without dementia, separate codes were created for those with and without dementia and compared. Codes were organised into descriptive themes by examining similarities and differences, then were developed into analytical themes in an inductive and deductive process by the first author, in discussion with a second researcher. The first author was informed by frameworks of personhood (Kitwood 1997) and the three ‘basic psychological needs’ of the self‐determination theory (SDT; Deci and Ryan 1985; Ryan and Deci 2000). Relationships between the themes were considered.

To address the second research question, data on methodological adaptations were extracted and summarised descriptively by the first author.

## 3 Results

### 3.1 Search Results

**FIGURE 1:**
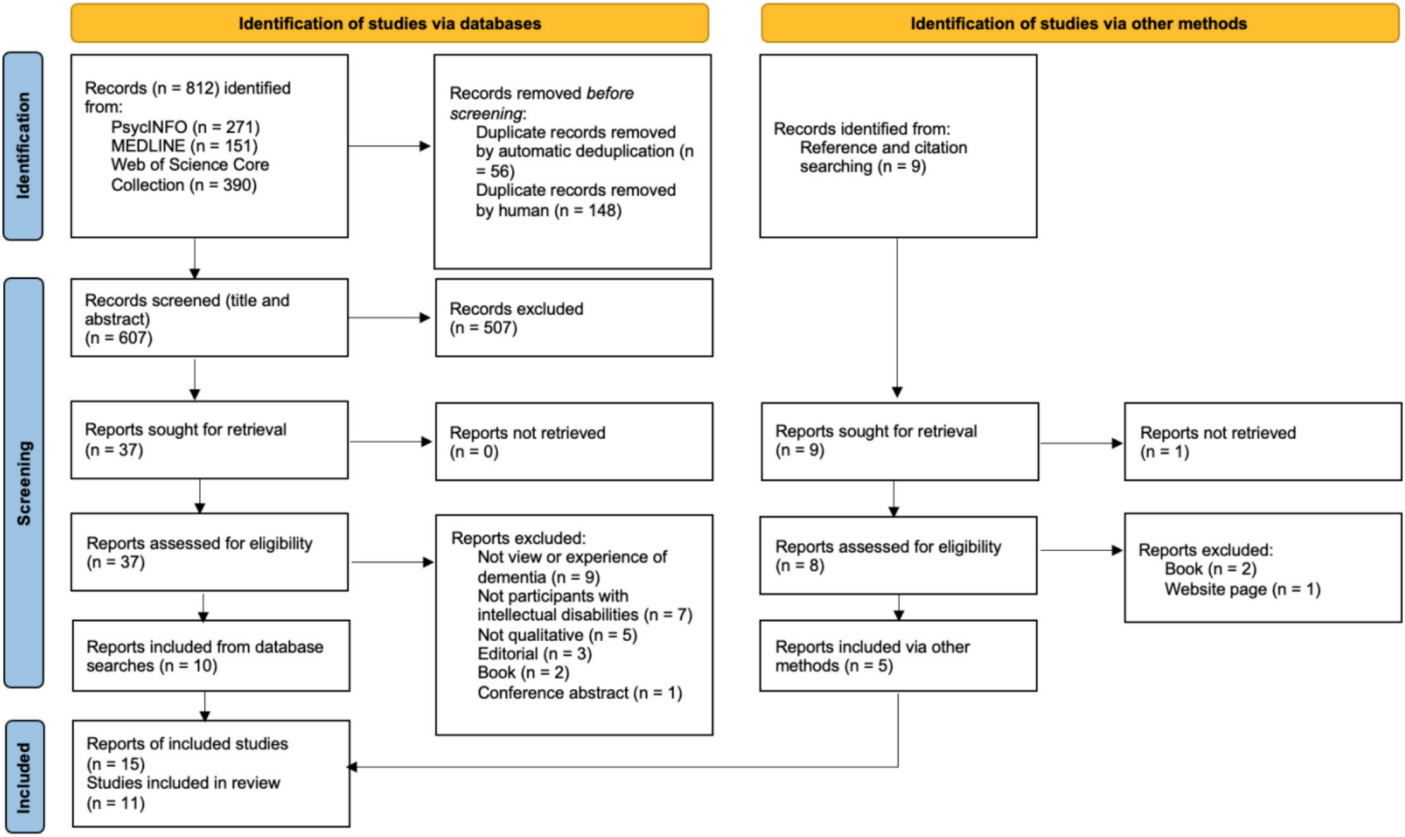
PRISMA flow diagram.

The study selection is outlined in Figure 1. Database searches conducted on the 14/09/2022 and repeated with the same search terms on 18/10/2024 identified 812 records. After duplicates were removed, 607 titles and abstracts were reviewed against the eligibility criteria and 37 reports selected for full‐text review. Ten met the full inclusion criteria. Five reports were identified through reference and citation searches giving a total of 15 reports on 11 studies. Three studies were presented in more than one report, hence were treated as one study (see Table 2). One study (Sheth et al. 2021) carried out a secondary analysis of the same data presented in another paper (Sheth 2019a). As the research question and analysis differed; these reports were therefore treated as separate studies. This resulted in 11 studies outlined in Table 2. There was a full agreement between the first author and second researcher on the included studies.

**TABLE 2:** Characteristics of included studies.

Author (year)	Number of participants country and setting	Age range sex ethnicity	Qualitative methodology and analysis	Key findings
Forbat and Wilkinson (2008)	With dementia = 8 (only data from 2 used) Without dementia = 8 UK, residential home	Not reported	Interviews and ethnographic observation Thematic analysis	People with IDD were told dementia diagnosis but had little understanding. People with ID can acquire knowledge about dementia including signs and symptoms and impact. Awareness of potential environmental changes and consequences but little communication about this.
Lloyd et al. (2007)	With dementia = 6 Without dementia = 0 UK, residential home for intellectual disabilities	Age = 49–59 M = 4 F = 2 White European = 6	Semi‐structured Interviews Interpretative phenomenological analysis	Roles and jobs are a sign of independence. People with ID view themselves as independent but opportunities diminish in dementia. People with IDD desire to maintain relationships and increasingly rely on others, especially staff and sometimes encounter relational difficulty. Some people with IDD recognise cognitive decline, more identify physical decline. Some experienced sense of loss and distress, some implicit evidence of coping strategies.
Lynggaard and Alexander (2004)	With dementia = 0 Without dementia = 4 UK, residential home	Age = 37–54 M = 2 F = 2 Ethnicity not reported	Semi‐structured interviews Data analysis method not reported	People with ID noticed changes in those who developed dementia. They did not attribute this to dementia or memory related changes but did following an intervention.
MacHale et al. (2024)	With dementia = 2 Without dementia = 3 Ireland, day centre for intellectual disabilities	Age = 54–64 M = 2 F = 3 Ethnicity not reported	Semi‐structured interviews Reflexive thematic analysis	Cognitive Stimulation Therapy is accessible for older adults with ID, including those with early dementia.
Manji (2008) Findings also reported in Manji and Dunn (2010)	With dementia = 4 Without dementia = 0 Canada, 1 in supported independent living, 2 in group homes, 1 in family home.	Age = 49–59 M = 1 F = 3 Ethnicity not reported	Observation Grounded theory	People with IDD experience losses in ability, home and community. They maintain selfhood with good health support decision‐making, self‐agency and autonomy. Good health support includes emotional support, particularly around grief. Maintaining connections with wider community activities and social lives is important. Staff empowered people with IDD to maintain selfhood, freedom and choice and involvement in community. Lack of staffing and funding restricted the quality of support.
Sheth (2019a) Findings also reported in Sheth (2019b)	With dementia = 4 Without dementia = 0 USA, community group homes for 6 or less.	Age = 45–61 M = 0 F = 4 White = 4	Nominal group technique analysed with thematic analysis. Ethnographic observations and unstructured interviews analysed using grounded theory approach and an ecological systems framework	Activity access is important including consistency, choice and agency Caregivers facilitate and limit choice and participation. Staff availability and relationship quality affects whether people ask for support. Positive social interactions facilitates participation, negative interactions are a barrier. Roles and responsibilities are important in day‐to‐day life including domestic tasks and relational roles Privacy and ability to be physically separate from others is important but difficult in shared accommodation. Good health and wellness facilitate participation, stress of self and others is a barrier.
Sheth et al. (2021) Secondary analysis of Sheth (2019a) data, participants and methodology are the same.	As above	As above	Ethnographic observations with unstructured interviews Thematic analysis used to analyse data	People experience many home moves, which can be positive (e.g., new friends and space) and negative (e.g., sadness, grief and lack of choice). Photos of people or places from the past help people engage with transition. Peer networks support transition but sometimes disrupted by changes in housing. Anticipation and threat of future transitions affects daily life. Fear of losing independence if not adhering to regulations or expressing negative emotions
Temple (2002)	With dementia = 2 interviewed Without dementia = 0 Canada, setting unclear	Age = 40–60 M = 1 F = 1 Ethnicity not reported	Semi‐structured interviews Data analysis method not reported	People with IDD can recognise changes in memory This is not always associated with sadness or concern but can be associated with positivity.
Watchman (2013) Findings also reported in Watchman (2016)	With dementia = 3 Without dementia = 0 UK, Intellectual disability group home (*n* = 1) Single tenancy with outreach support (n = 1) Generic care home for older people (n = 1)	Age = 47–60 M = 1 F = 2 Ethnicity not reported	Longitudinal ethnography Thematic analysis	Sense of self is maintained for people with IDD and is not dependent on verbal ability. This is displayed through: ability to reflect own views; insight into physical mental or emotional attributes and characteristics and through social interactions A lack of shared diagnosis led to more fear. People experience exclusion and isolation, and losses of relationships, participation in social activities and decision making.
Watchman et al. (2020) Findings also reported in Watchman et al. (2021)	Termed ‘co‐researchers’ in this paper: With dementia = 1 Without dementia = 3 UK, setting of co‐researchers not reported.	Not reported	Photovoice methodology Thematic analysis	People with ID had limited knowledge of dementia prior to the study. Dementia was associated with fear and uncertainty and sense of loss and unknown, compounded by dementia not being explained. Friendship and support from peers, family and especially staff seen as important Transitions in accommodation can hinder support and friendship. People have questions about the future and want to be involved in future care decisions; accessible information is important for this.
Watchman et al. (2024)	With dementia = 1 Without dementia = 4 UK, setting not reported.	Age = 57–69 M = 3 F = 2 White = 5	Semi‐structured, narrative life story interviews Thematic analysis	People with ID affected by dementia can maintain intimate relationships, though the emotional impact of this was noted with a fear of separation and need to plan for the future. There is a need for support staff to have specific knowledge about ID and dementia as well as a couples' life story. Partners actively took on caring roles, challenging views of being solely care‐receivers, but expressed how this can be scary and worrying.

### 3.2 Description of Studies

Qualitative data were reported for 47 participants with intellectual disabilities (25 with dementia and 22 without) with an age range of 37–64 and a mix of genders, although some studies did not report this data. Seven studies did not report ethnicity. Of those that did, all participants were ‘White’ or ‘White European’. All studies were from the United Kingdom, Ireland or North America. The results of the CASP checklist are displayed in Table 3 with studies scoring between 7 and 10 of a possible 10. Six studies did not report on researcher role and two studies did not report enough detail on recruitment or data analysis.

**TABLE 3:** CASP quality assessment summary.

Author (year)	Aims stated and justified	Qualitative methodology appropriate	Research design	Recruitment strategy	Data collection	Researcher role	Ethics	Data analysis	Statement of findings	Value of research	CASP score
Forbat and Wilkinson (2008)	Yes	Yes	Yes	No	Yes	No	Yes	No	Yes	Yes	7
Lloyd et al. (2007)	Yes	Yes	Yes	No	Yes	No	Yes	Yes	Yes	Yes	8
Lynggaard and Alexander (2004)	Yes	Yes	Yes	Yes	Yes	No	No	No	Yes	Yes	7
MacHale et al. (2024)	Yes	Yes	Yes	Yes	Yes	No	Yes	Yes	Yes	Yes	9
Manji (2008)	Yes	Yes	Yes	Yes	Yes	Yes	Yes	Yes	Yes	Yes	10
Sheth (2019a) Sheth (2019b)	Yes	Yes	Yes	Yes	Yes	No	Yes	Yes	Yes	Yes	9
Sheth et al. (2021)	Yes	Yes	Yes	Yes	Yes	No	Yes	Yes	Yes	Yes	9
Temple (2002)	Yes	Yes	Yes	Yes	Yes	Yes	Yes	Yes	Yes	Yes	10
Watchman (2013)	Yes	Yes	Yes	Yes	Yes	Yes	Yes	Yes	Yes	Yes	10
Watchman et al. (2020)	Yes	Yes	Yes	Yes	Yes	Yes	Yes	Yes	Yes	Yes	10
Watchman et al. (2024)	Yes	Yes	Yes	Yes	Yes	Yes	Yes	Yes	Yes	Yes	10

### 3.3 Perspectives of Dementia: Thematic Synthesis Findings

Three analytical themes were identified with five further subthemes: dementia is associated with loss (loss of ability and loss of relationships and connection); the importance of maintaining a sense of self (through maintaining choice, maintaining competence and maintaining relational connection) and support counteracts loss. Data from those with and without dementia identified similar experiences, so codebooks were amalgamated. The results outline where a theme related to just those with dementia, without dementia or both groups.

### 3.4 Dementia Is Associated With Loss

Experiences of loss were in the accounts of participants with and without dementia (nine studies). Loss of ability, and loss of relationships and connection were two key areas.

#### 3.4.1 Loss of Ability

Thirteen participants with intellectual disabilities and dementia had not been told about their diagnosis of dementia (four studies), and participants who had been told did not remember this. Some did not recognise any cognitive difficulties; however, several were aware of changes.

Participant They tell me and I keep forget, yeah. Yeah, errm they told me Julie, I forgot the name, Julie. I can't think, yeah. (Lloyd et al. 2007)

Participants with intellectual disabilities and dementia seemed more aware of their physical compared to cognitive decline. Participants were also aware of the impact of changes on their life, and no longer being able to do things they previously could do.

Participant Hard to put away clothes. Hard on my legs to go up and down stairs. (Temple 2002)

Distress and frustration related to cognitive changes of dementia and fear for the future were present in accounts of those with and without dementia.

Participant Yeah (pause), I don't want to pass away. I don't want to get old. I don't want to go to heaven. I can't, I can't lose it. The way things are. (Lloyd et al. 2007)

Dementia had not been explained to participants without dementia, leading to fear. Participants without dementia noticed changes in the behaviour of people with intellectual disabilities and dementia, though did not attribute this to dementia. They were better able to understand dementia and attribute behaviour changes to this once it was explained and discussed with them.

Researcher and participant [Participants] had little or no understanding that the changes in their housemates' behaviour were the result of [dementia] … one person said that the housemates ‘should pull themselves together’, and another said that ‘they are lazy, like a baby’. (Lynggaard and Alexander 2004)

#### 3.4.2 Loss of Relationships and Connection

Participants with and without dementia spoke about loss of relationships with peers, family and staff due to dementia, including through the death of peers with dementia.

Researcher ‘Shortly after eating, Donna began crying and pointing to the picture of [name] by the front door.’ it was obvious that Donna was expressing grief for a dear friend and housemate with dementia who had passed on. (Manji 2008)

Dementia‐related accommodation changes led to losses of peer and staff relationships. Sometimes participants were facilitated to maintain relationships or coped by forming new friendships.

Researcher and participant when seeing an old friend at a crafting group. Karen reached out to hold their hand and commented, “It's not the same without you” (Sheth et al. 2021)

Relational disconnection was feared by participants and present in accounts, when participants with intellectual disabilities and dementia were not aware or did not acknowledge their difficulties when it was evident to others.

Participant What if my behaviour to my wife changes, how will we cope? (Watchman et al. 2024)

Researcher Generally, participants commented that it had become much harder to have conversations with the two people with dementia and that there were many more arguments in the house. (Lynggaard and Alexander 2004)

### 3.5 The Importance of Maintaining a Sense of Self

Many accounts referenced the importance of maintaining a sense of self (nine studies) through the interconnected subthemes of maintaining choice, competence and relationships.

#### 3.5.1 Maintaining Choice

The ability of participants with intellectual disabilities and dementia to make choices was evident in many domains including choosing food, clothes or activities, whether to engage or have time alone, what to buy and how to decorate a room.

Researcher Staff is trying to find out what Jim would like to eat today. She signs, “Chinese Rice?” Jim gets excited and nods his head smiling. Similarly, I observed individuals deciding what to wear, which bathroom to use, when to go to bed, and what to do with their time. (Manji 2008)

The importance of choice about the future was also evident in accounts from those with and without dementia.

Participant I think one of the things that's important for them to know is to think about what is going to happen in a few months or years and what they need to do to the house—who can help with that? (Watchman et al. 2020)

Participants communicated choices in a variety of ways; verbally, signing, or through nonverbal behaviour, for example, choosing to not engage in an activity when needing to be present.

Researcher Karen initially did not engage in this structured programming, notably walking off from the main activity. When told several times that she needed to come join the group, she walked over at sat with the other participants, but did not engage. (Sheth et al. 2021)

#### 3.5.2 Maintaining Relational Connection

The importance of relational connection with others was identified by those with and without dementia. Participants with dementia valued time with their partner, families and friends, even when difficult; with peer relationships having a reciprocal nature and being outlets for fun and support.

Participant we'd have a cuddle and then we'd be all right together. (Watchman et al. 2024)

Participant I help out when people need help […] [friend] help me out and I help her out. (Sheth 2019b)

Participants found ways to maintain connection in a way that was unaffected by dementia, including imagined interactions and staff fulfilling the role of friends and carers.

Participant Charlie: Louise and Jane (residential home carers), they’re my friends. (Lloyd et al. 2007)

Researcher and Participant Most clients identified the facilitators of the group as their friends, ‘you (will) always be my friend, my best friend’. (MacHale et al. 2024)

#### 3.5.3 Maintaining Competence

Participants' ability and desire to maintain competence in relational abilities, areas of interest, or ability to carry out chores was present.

Participant Interviewer: Can you remember things ok? Charlie I can yes. But it’s a bit bad. But I know this (spells out own name with fingers). (Lloyd et al. 2007)

Where there was more significant loss of ability participants took on simple tasks as their role or job to complete.

Researcher JF spent a good deal of time sorting Christmas cards back and forth into piles. (Temple 2002)

In the latter stages of dementia, competence to communicate and maintaining specific interests were present. Maintaining the appearance of competence in the absence of ability was also present, though unclear whether due to lack of insight or a conscious strategy to maintain a sense of self.

Researcher and participant As time passed, and Hannah’s dementia progressed, I was unsure if she would remember or maintain her interest in bags although this proved unfounded. Researcher: Hannah would you like the bag? [Field notes Hannah inaudible – high pitched but not distressed, calm and smiles then screeches loudly, takes bag, strokes it]. Researcher: [Calming voice] That’s okay. (Watchman 2013)

### 3.6 Support Counteracts Loss

Participants' accounts highlighted the importance of others in counteracting the losses associated with dementia and supporting maintenance of sense of self (seven studies). Choice was facilitated when others empathised and understood wishes and desires, and when others had a lack of knowledge of a person's abilities and wishes, this was a barrier to choice.

Researcher Jenny goes in her bedroom … When staff asked her if she wanted to go to bed, she said ‘Nein.’ Then staff asked her if she wanted to go to the living room. She said, ‘Nein.’ Then staff asked her if she needed a hug. She said, ‘Ha!’ Staff gave her a hug, and Jenny crawled into bed. Staff said Jenny had decided to go to bed early today. (Manji 2008)

Support from others helped participants maintain competence and independence, with staff taking time to include and support people with dementia to carry out tasks rather than doing it for them. This seemed to give participants a sense of achievement. Support also helped participants maintain relational connection and lack of awareness of relational needs hindered this.

Participant I don't need any other help. Louise (staff caregiver) has to do my bed for me sometimes. But I help as well. (Lloyd et al. 2007)

**FIGURE 2:**
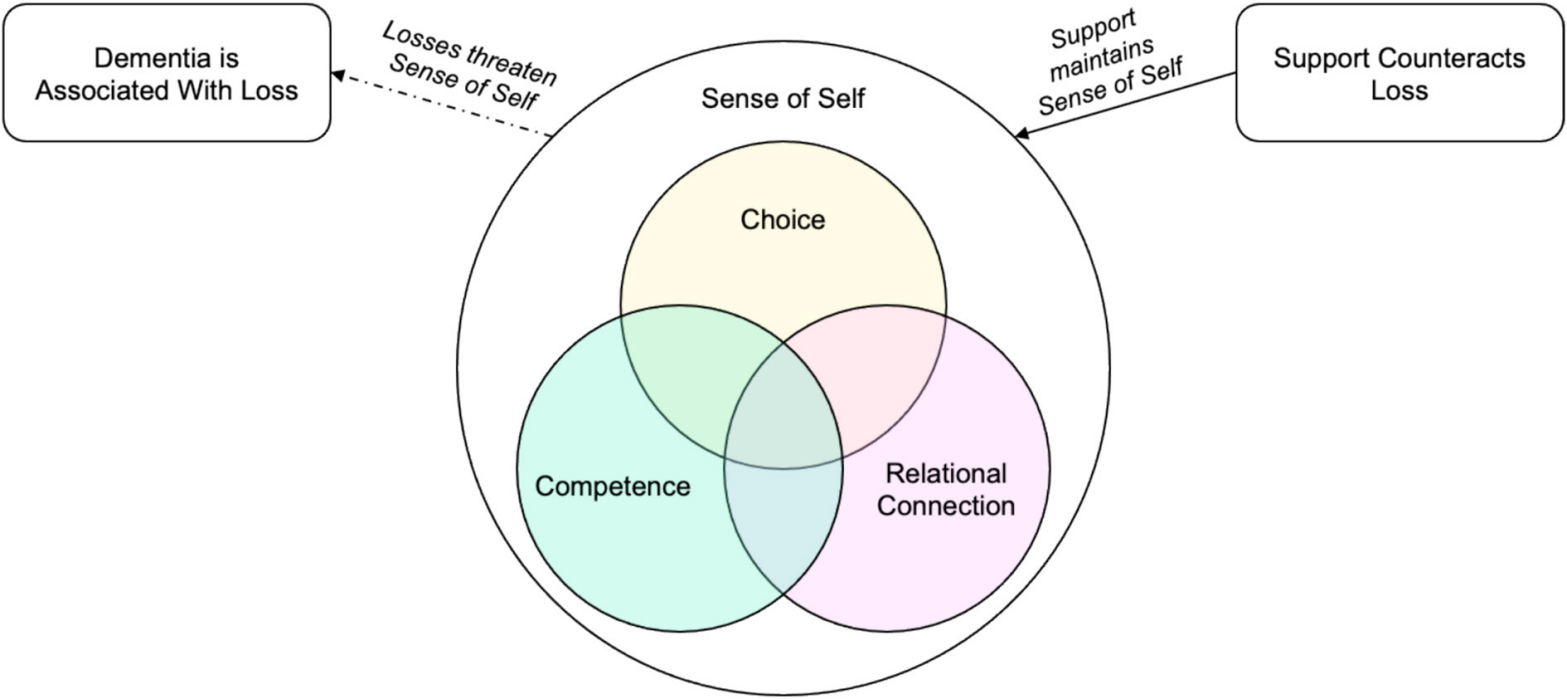
Diagram of how the analytical themes relate to each other.

As analytical themes were developed, relationships between them were also considered and they seemed connected rather than isolated concepts (Figure 2). Losses associated with dementia seemed to threaten sense of self. Conversely, support from others seemed to maintain the sense of self and counteract the effects of the dementia. The three themes of choice, competence and relational connection, developed deductively from the SDT, had a degree of overlap, for example, participants demonstrated choice and competence in relationships.

### 3.7 Methods to Include People With Intellectual Disabilities and Dementia in Qualitative Research Findings

Studies included several adaptations to involve participants with intellectual disabilities and dementia. Four highlighted this explicitly: one focusing on overcoming methodological and ethical challenges (Watchman 2016), one including guidelines for research with this population (Manji 2008), one describing the adaptation of Nominal Group Technique for use in this population (Sheth 2019a) and one describing the use of life story interviews, which was used with one participant with intellectual disabilities and dementia (Watchman et al. 2024).

As participants did not necessarily know their dementia diagnosis, studies avoided the terms ‘dementia’ or ‘Alzheimer's’ in interactions and patient facing paperwork (three studies) and asked carers about awareness of diagnosis in advance (Sheth 2019a).

Using pictures on documents aided accessibility and facilitated consent (four studies). Consent was seen as an ongoing process (two studies), with assessment of participants' wish to remain involved at each research activity. This included paying attention to communication through body language and non‐verbal cues.

Studies simplified language, used both written and verbal communication, reframed questions and used examples. Large fonts with pictograms alongside words were used to facilitate communication (Sheth 2019a). One study used sign language (Manji 2008) with another mentioning it as a helpful approach (Lloyd et al. 2007).

To address limited verbal data, researchers collected and triangulated between different types of data. For example, case records and/or interviews with carers (four studies) or interviews and observations (three studies). Field notes enabled nonverbal data to be captured when verbal communication was limited.

Longitudinal approaches also enhanced the quality of the data (two studies and balanced gathering data with not being overly intrusive (Watchman 2013).

Allowing adequate time for participants to respond was noted, with relevant responses given a while after questions being asked (Watchman 2013). The same study noted the use of touch and music as means of communication and the use of objects of reference.

Other considerations included meeting with participants prior to interviews to discuss the process and develop rapport (two studies), and considering practice sessions (Sheth 2019a). Most studies did not include carers in interviews; however, one noted that carers helped with prompts to elaborate, rephrasing, or clarifying (Sheth 2019a).

One study used Nominal Group Technique to facilitate engagement in the research (Sheth 2019a). The group technique enabled participants to benefit from others' ideas. However not all participants were able to engage in this methodology, and, although pictograms were used in places, the methodology still relied heavily on written text. Another study used life story interviews using pictures as visual guides to the interview as well as photographs brought by the participants (Watchman et al. 2024).

## 4 Discussion

This review provides insight into the views and experiences of dementia from the perspective of people with intellectual disabilities both with and without dementia. Perhaps the most striking finding is the similarities in perspectives of people with intellectual disabilities included in this review and findings from dementia research in the general population (Birt et al. 2020; Bolt et al. 2022). Dementia is associated with a sense of loss and fear for the future. Maintaining choice, competence and relational connection sustains sense of self, and support from others is a facilitator or barrier to that. People with intellectual disabilities and dementia were more aware of physical than cognitive decline, aligning with ageing research in the intellectual disability population (McCausland et al. 2021). This review additionally found participants were able to acquire knowledge about dementia; perhaps there is a lack of discussion rather than a lack of ability to understand.

The findings also identify many similar themes to those of the review including proxies (Jacobs et al. 2023), however, differ in the part that this review did not highlight explicitly the role of marginalisation and stigma. Perhaps this is where proxies give wider context and language to the experiences of this population.

Tom Kitwood (1997) posited that other people are crucial in the maintenance of sense of self in dementia and the important role of others is also evident in this review. As verbal or conscious communication declines, there is an increasing need for carers to understand the unconscious and bodily communication of wishes and desires (Wyatt 2021). However, recent literature has challenged the sense of passivity in Kitwood's model, positioning people with dementia as having agency in their social world (Birt et al. 2020). The relationship between the findings in Figure 2, highlighting the need for competence as well as support from others, suggest that perhaps it is not an ‘either/or’ of agency or passivity, but rather a ‘both/and’; people with intellectual disabilities and dementia can have agency, and others can contribute to this.

Methodological adaptations facilitated participation of those with intellectual disabilities and dementia in research, mirroring findings in the dementia literature (Wilkinson 2002). Studies employed multiple adaptations, and the impact on researcher time, capacity and resources is not insignificant. Therefore, it seems important in this field of research to set up and plan research well.

The studies were limited by having a small number of participants in solely white‐western populations. The methodology of this review was limited by having a sole researcher creating codes rather than two and for the synthesis of methodologies used for participants with intellectual disabilities and dementia; the small number of studies limited the ability to use more rigorous synthesis methodologies. As further research is undertaken with this population, this can be addressed.

### 4.1 Implications

The findings highlight that people with intellectual disabilities can understand dementia, but that it is not always discussed. One of the clinical implications of this research is therefore the need to ensure dementia diagnoses are communicated in a sensitive way to individuals as well as the need to raise awareness amongst individuals with intellectual disability and support them to make choices in advance about their wishes.

This review also highlights how carers should try to offer opportunities for choice, competence and connection for people with intellectual disabilities and dementia and try to support this where needed as well as encouraging agency. Training and raising awareness of staff and carers is likely an important factor in this, particularly around supporting choice and agency in big and small decisions. Given the emotional impact of loss, there is potential to minimise this by maintaining connections with previous friendships or facilitating the creation of new friendships.

More high‐quality studies are required. Further research in non‐western cultures is particularly needed, with a need for improved reporting, including sampling methods, demographic data, data analysis and author reflexivity. This review goes some way to addressing the need for identifying data collection methodologies (Watchman et al. 2019), however more research exploring methodologies is needed. Further research exploring the use of visual communication aids such as Talking Mats is an interesting area of future research given encouraging use in similar populations (Murphy et al. 2010; Murphy and Cameron 2008). Adaptations highlighted in this review should be used, built upon, and tested to improve inclusion of this population in research.

## Conflicts of Interest

The authors declare no conflicts of interest.

## Supporting information


**Data S1** Supplementary Information.

## Data Availability

Data sharing is not applicable to this article as no new data were created or analyzed in this study.
