# Supplementary material for: Views and Experiences of Dementia in People With Intellectual Disabilities: A Systematic Review of Qualitative Research
Source: J Intellect Disabil Res. 2025 Mar 16;69(7):533–45. doi: 10.1111/jir.13227 (PMC12198092; doi:10.1111/jir.13227)
Supplement: Supplementary file 1 — Data S1 Supplementary Information. [file JIR-69-533-s001.docx]

**Supplementary Information 1**

Below is the search strategy for PsycINFO and Medline and following this is the search strategy for the Web of Science.

1. exp Intellectual Development Disorder/

2. (Intellectual Disabilit* or Learning Disabilit* or Down? Syndrome or Learning Difficult*).mp. [mp=title, abstract, heading word, table of contents, key concepts, original title, tests & measures, mesh word]

3. 1 or 2

4. exp Dementia/

5. (Dementia or Alzheimer* or Major Cognitive Disorder or Memory).mp. [mp=title, abstract, heading word, table of contents, key concepts, original title, tests & measures, mesh word]

6. 4 or 5

7. exp Client Participation/

8. exp Client Attitudes/

9. (Opinion* or View* or Perspective* or Attitude* or Experience* or Voice* or Perception* or Participation or Involve* or Engage* or Input or Contribut*).mp. [mp=title, abstract, heading word, table of contents, key concepts, original title, tests & measures, mesh word]

10. 7 or 8 or 9

11. exp Qualitative Methods/

12. exp Interviews/

13. (Qualitative* or interview* or focus group or participatory action research or photovoice or talking mat* or easy?read or Co?production or case study).mp. [mp=title, abstract, heading word, table of contents, key concepts, original title, tests & measures, mesh word]

14. 11 or 12 or 13

15. 3 and 6 and 10 and 14

Below is the search strategy for the Web of Science:

(((ALL=(“Intellectual Disabilit*” OR “Learning Disabilit*” OR “Down*Syndrome” OR “Learning Difficult*”)) AND ALL=(Dementia OR Alzheimer* OR “Major Cognitive Disorder” OR Memory)) AND ALL=(Opinion* or View* or Perspective* or Attitude* or Experience* or Voice* or Perception* or Participation or Involve* or Engage* or Input or Contribut* or Co*production )) AND ALL=(Qualitative* or interview* or focus group or “participatory action research” or photovoice or “talking mat*” or “easy*read” or “case study”)
